# Effects of school-based mindfulness intervention on health-related quality of life: moderating effect of gender, grade, and independent practice in cluster randomized controlled trial

**DOI:** 10.1007/s11136-021-02868-4

**Published:** 2021-06-24

**Authors:** Maarit Lassander, Mirka Hintsanen, Sakari Suominen, Sari Mullola, Tero Vahlberg, Salla-Maarit Volanen

**Affiliations:** 1 Faculty of Medicine, Department of Psychology and Logopedics, University of Helsinki, Parrulaituri 16 D 62, 00540 Helsinki, Finland; 2 Unit of Psychology, University of Oulu, Oulu, Finland; 3 Department of Public Health, University of Skövde, Skövde, Sweden; 4 Department of Public Health, University of Turku, Turku, Finland; 5 Folkhälsan Research Center, Helsinki, Finland; 6 Faculty of Educational Sciences, University of Helsinki, Helsinki, Finland; 7 Department of Biostatistics, University of Turku, Turku, Finland; 8 Teachers College, National Center for Children and Families (NCCF), Columbia University, New York, NY USA; 9 Faculty of Medicine, Department of Public Health, University of Helsinki, Helsinki, Finland

**Keywords:** Health-related quality of life, Mindfulness, School interventions, Children, Youth

## Abstract

**Object:**

We investigated the impact of a school-based 9-week mindfulness program vs*.* active control program (relaxation) and inactive control group on children’s self-reported Health-Related Quality of Life (HRQoL) moderated by gender, grade, and independent practice.

**Method:**

In total 3519 (50/50% boys/girls) Finnish students aged 12–15 years from 56 schools were randomized into mindfulness intervention, active, and inactive control groups. HRQoL was measured at baseline, at 9 weeks, and at 26 weeks and analyzed with multilevel linear modeling.

**Results:**

Significant improvement on HRQoL was found (*β* = mean difference) (*β* = 1.587, 95% CI 0.672–2.502, *p* < 0.001) after 9 weeks and at 26 weeks of follow-up among students in the mindfulness group as compared to the active control group. Moderating effects on HRQoL were found for gender, grade, and independent practice: girls, 7th and 8th grade students, and students with regular independent mindfulness practice benefited most.

**Conclusions:**

Gender and developmental stage may moderate the effects of mindfulness interventions on HRQoL and offer guidance in designing effective promotive interventions for children and adolescents.

**Trial Registration Information:**

Healthy Learning Mind—a school-based mindfulness and relaxation program: a study protocol for a cluster randomized controlled trial (RCT) ISRCTN18642659 retrospectively registered on 13 October 2015. The full trial protocol can be accessed at http://rdcu.be/t57S.

**Supplementary Information:**

The online version contains supplementary material available at 10.1007/s11136-021-02868-4.

## Introduction

Children’s satisfaction or happiness in various life domains can be assessed with Health-Related Quality of Life (HRQoL) measures [43, 46]. HRQoL is often applied as an outcome measure for children’s immediate life experiences in intervention evaluation and typically includes domains, such as physical and psychological well-being, family/peer support, and well-being in school [46]. However, there are very few studies examining how to generally improve quality of life without linking it to functional impairment or illness following medical or psychosocial interventions [50]. There is also a definitive lack of studies that would examine the impact of mindfulness-based intervention on HRQoL incorporating a controlled experimental design with follow-ups beyond the end of the intervention.

For the past decade, mindfulness-based school interventions have been found to produce small (effect size *g* = 0.22–0.40) or mixed results [23, 26] in the well-being of children and adolescents on a variety of measures, such as cognition, stress, anxiety and depression, and psychological well-being [8, 17, 18, 42]. This lack of consistency (for reviews see [10, 33]), raises questions about the potential moderators for observed effects [19, 41].

### Health-related quality of life and its moderators

HRQoL is a subjective and multidimensional concept [46] across multiple systems of function (family, peer groups, school, community). These dimensions affect or are affected by health and provide generic understanding of subjective health and well-being [15, 40]. HRQoL is particularly well-rounded measure for children and adolescents, as it takes into account the spheres of individual experience, family, and school environment [37, 47, 50].

Previous research has found consistent gender differences suggesting that girls have poorer HRQoL in general compared to boys. Boys report higher HRQoL, namely, on the dimensions of physical and psychological well-being, and parents’ relation and autonomy [34, 39]. There is an established, cross-cultural effect of HRQoL decreasing with age [36, 39].

### Mindfulness practice and its moderators

Mindfulness consists of self-regulation of attention maintained in immediate experience and practicing the qualities of curiosity, openness, and acceptance directed toward these experiences [32, 41]. Research on mindfulness suggests that females, both adults and adolescents, in general benefit more from mindfulness interventions, especially in terms of psychological well-being [12, 13, 27]. As the academic pressure and social media use place increasing demands to students mental health [28]; [31], school-based mindfulness programs teach directing attention and encourage acceptance toward emerging emotions [21], higher self-compassion, and self-acceptance [4, 7, 24]. For adolescents, mindfulness practice may also lessen the detrimental self-focus in challenging situations [5] and support adaptive development [2].

There is still limited evidence on the moderating effect of age on the effects of mindfulness practice [10]. One study [44] found that 13-year-olds (age when Finnish students transfer from primary to middle school) seemed to benefit more from mindfulness over relaxation compared to 12-year-olds and 14–15-year-olds in terms of socioemotional functioning. A recent meta-analysis suggests that interventions delivered during late adolescence (among 15–18-years-old) had the largest effects (in comparison with ages 6–14) on mental health and well-being outcomes, whereas gender differences were not found [10].

What comes to independent mindfulness practice, so far, we know very little about its significance for children and adolescent well-being. Some research suggests [44] that among 12–15-year-olds regular independent practice (almost daily) enhanced the intervention effects, i.e., increased resilience and socioemotional functioning. Among adults, in turn, a recent meta-analysis [38] found a small but consistent association between more frequent formal practice and positive intervention outcomes, presenting a wide range of psychological measures.

### Relaxation as an active control

Relaxation training has often been applied as an active control intervention when examining the effects of mindfulness [25]; [30]. Both relaxation and mindfulness training reduce anxiety and may increase positive mood states, but mindfulness-based interventions may also reduce negative reactivity to ruminative thoughts [16, 22].

### Present study

In the current cluster randomized study, we examine effects of mindfulness-based intervention (stop and breathe) [20] as compared with standard relaxation program “Relax” and inactive control group on HRQoL moderated by grade, gender, and independent practice. We hypothesize that (1) mindfulness intervention improves the HRQoL of 12–15-year-old children (6th–8th grades) [8, 17, 49]; (2) girls will benefit more from the intervention compared with boys [26, 44]; (3) adolescents starting a new school phase will benefit more from intervention compared to other age groups [44],and (4) more intensive independent practice is associated with higher intervention effects in terms of HRQoL [38, 49]. To the best of our knowledge, this is the first controlled study with follow-up continuing past the immediate post-intervention assessments exploring the effects of a mindfulness-based intervention on HRQoL. We also address current gaps in the literature, regarding how to improve general quality of life in adolescent population and how the moderating effects of age and independent practice can affect the outcomes.

## Methods

The study is cluster randomized controlled trial (RCT). Participating schools were randomly allocated to an intervention, active control (relaxation), or inactive control group [45].

### Participants

Study data were gathered in 2014–2015. Participants were invited from 247 schools in 14 cities/municipalities in Southern Finland. Among contacted schools 56 (24%) chose to participate (Fig. S1). Participants were sixth, seventh, and eighth graders (age 12–15) in Finnish comprehensive school.

### Procedure

The ethical review board of the University of Helsinki (approval 1/2014) reviewed the study plan. A consent to participate was requested from all headteachers for randomization, intervention, and data collection. A written informed consent was requested from all students and their parents for data collections and the study was conducted according to the Helsinki Declaration. Participants could withdraw their participation at any point without giving any reason. Data handling and analyses were performed according to the EU General Data Protection Regulation, and personal identification of the participants was removed from all data.

The participating schools were first recruited and then randomized before the baseline data collection. The schools were randomly assigned to intervention schools (*N* = 25 schools with 94 classes), active control schools (*N* = 24 schools with 85 classes), and inactive control schools (*N* = 7 schools with 31 classes). Inactive control group was included in study design to ensure comparability with other mindfulness intervention studies, which usually only include an inactive control group. The main comparison was done with the active control group, ensuring that we can measure non-specific changes due to being part of an intervention. Whole class participated in the designated intervention/control. Classes in the intervention and in the active control groups were informed about participation in a 9-week program called “Skills for Wellbeing,” taught by trained facilitators. The participants were blinded as to whether they were selected to intervention or active control program.

### Mindfulness intervention

The intervention, 9-week mindfulness program Stop and Breathe [20] began after baseline measurements. This program includes nine weekly 45-min group sessions and short home practices (the recommended amount of practice being 5–6 times per week, approx. 3–15 min at a time) and is designed to improve emotional awareness, sustained attention, and attentional and emotional regulation. Preliminary research suggests it to be effective in decreasing depressive symptoms, lowering stress, and enhancing psychological well-being [29]. Sessions started with a psychoeducational introduction to the themed lesson (e.g., directing attention, experiencing difficult thoughts, and difficult emotions), including short formal or informal practices, group discussion, and ending with a longer practice. Mindfulness home practices were available to download from the course website [45].

### Control groups

Active control group underwent a 9-week standardized relaxation program “Relax.” The aim of the Relax program is to enhance students’ relaxation skills and holistic well-being. The frequency and duration of the weekly sessions (i.e., dose) of the Relax program is equal to the mindfulness program, including nine weekly 45-min group sessions and home practices (the recommended amount of practice being 5–6 times per week). The sessions consisted of psychoeducational presentations relating to well-being (e.g. stress, sleep, nutrition), relaxation exercises (e.g., progressive muscle relaxation, calming breathing, and visualization), pair and group discussions, and group assignments.

In the inactive control group, the participants followed normal school curriculum without any interventions. All groups filled in the same research questionnaires at baseline, at 9 weeks, and at 26 weeks of follow-up.

### Measures

Questionnaires and other tests were completed as a part of the RCT in 2014–15. Questionnaires were filled in at baseline, 9 weeks, and 6 months during the normal school hours.

To measure Health-Related Quality of Life we chose KINDL-R, a generic instrument for assessing HRQoL in children and adolescents aged 3–17 [39]. The questionnaire has been developed based on interviews with children and adolescents to reflect their own priorities. KINDL-R consists of 5 domains (physical and psychological well-being, self-esteem, family, friends, and school) each indicated with 5 self-report items assessed on a 5-point likert scale from 1 (strongly disagree) to 5 (strongly agree; transformed score range 0–100) and giving the time frame of past week. Higher scores indicate better HRQoL. In the current study baseline Cronbach’s alpha coefficient of KINDL-R was 0.89.

The amount of intervention specific independent practice was self-reported at follow-up questionnaires. The respondents were divided into four groups: high frequency practice (nearly every day, *n* = 46), moderate frequency practice (once a week, *n* = 55), low frequency practice (once or twice a month *n* = 104), and infrequent practice (only few times, *n* = 733).

### Data analysis

The sample size was estimated to detect the mean difference of 0.2 standard deviation units (effect size = 0.2) on main outcomes between intervention and control groups with 80% power and the two-tailed 5% level of significance. The clustering of outcomes within schools was taken into account, assuming an intra-cluster (intra-school) correlation coefficient of 0.03 and assumed that on average 60 children in each school will complete the study. The required sample size was estimated to be 1090 children per group, and allowing for about 10% dropout rate, the study required 1200 children per group and total of 2400 children to be recruited. Using the same assumptions to detect the mean difference of 0.3 standard deviation units (effect size of 0.3) between intervention and non-treatment group, the required sample size was estimated to be 486 children per group, and allowing for about 10% drop-out rate, the study required 540 children in the non-treatment group.

The effect of intervention on HRQoL was analyzed with multilevel models to account for the clustered nature of the data. Four-level models with time at level 1, student at level 2, students in a particular classroom at level 3, and school at level 4 were fitted. Intra-class correlation (ICC), which is the proportion of the total variance explained by each level, was calculated for student, classroom, and school level. ICCs at the school level were low (ICC < 0.01) and variances of random intercepts for school level were non-significant, despite the school level being the unit for randomization. Thus, school-level variance was excluded from the final multilevel models and a three-level model with time at level 1, student at level 2 (ICC = 0.62), and classroom at level 3 (ICC = 0.06) was used. In addition to variance components at the classroom level and student level, the covariance between random components was also included in the models (not shown in the tables), if estimable. Maximum likelihood estimation was used to obtain unbiased and efficient parameter estimates for data with missing values in the follow-up measurements.

The modifying effect of gender and grade, i.e., whether the intervention effect was different depending on gender or grade, was analyzed with multilevel modeling. The modifying effect of independent practice (all self-motivated practice taking place outside the program sessions) underwent similar analysis.

Multilevel linear models included the main effects of group, time, gender, and grade. The intervention effect was examined by interaction terms between group (intervention vs. active control and intervention vs. inactive control) and time (9 weeks vs. baseline and 26 weeks vs. baseline). To show positive intervention effects the estimates for interaction effects (group × 9 weeks and group × 26 weeks) were required to be positive for HRQoL. Interaction effects indicated the change in HRQoL in the intervention group compared to the active control and non-treatment groups. To analyze the modifying effect of gender and grade, i.e., whether the intervention effect (intervention vs. active control and intervention vs. non-treatment) was different depending on gender or grade, the second-order interaction term group × grade × time or group × gender × time was entered to the model. To examine whether the intervention effect differed depending on the continuing independent practice intensity after the intervention compared to the active control and non-treatment groups, the interaction term practice intensity group × time was entered to the model.

Multilevel linear modeling was done with MLwiN Version 2.35 (Centre for multilevel modelling, University of Bristol) and other analysis with the SAS System for Windows 9.4 (SAS Institute Inc., Cary, NC). Two-sided statistical tests with a 5% significance level were used, and no adjustments were made for multiplicity.

## Results

### Sample characteristics

Out of 3519 students participating in this study 2754 students provided at least one measurement of quality of life at baseline, at 9 weeks, or at 26 weeks of follow-up, 1220 in intervention, 1181 in active control, and 353 in inactive control group (see Table 1).

**Table 1:** Descriptive statistics for KINDL-R subscales at baseline, 9 weeks, and 26 weeks for all students and by gender

Intervention group							Active control group						Non-treatment group					
Scale variable	*N*	Baseline Mean (SD)	*N*	9 weeks Mean (SD)	*N*	26 weeks Mean (SD)	*N*	Baseline Mean (SD)	*N*	9 weeks Mean (SD)	*N*	26 weeks Mean (SD)	*N*	Baseline Mean (SD)	*N*	9 weeks Mean (SD)	*N*	26 weeks Mean (SD)
All students																		
Physical well-being	1203	69.56 (21.95)	1156	70.91 (20.86)	949	71.88 (20.26)	1170	70.41 (21.53)	1095	70.53 (20.37)	994	71.88 (20.91)	353	60.93 (22.39)	319	73.01 (20.10)	305	71.83 (20.75)
Emotional well-being	1220	75.85 (15.74)	1152	75.88 (15.66)	957	75.95 (15.85)	1181	76.42 (15.31)	1104	74.20 (16.29)	1012	75.53 (16.54)	352	75.37 (15.62)	320	76.11 (16.41)	306	75.63 (15.60)
Self-esteem	1216	62.84 (20.04)	1155	65.03 (20.16)	953	65.57 (19.31)	1179	62.14 (19.92)	1102	63.84 (19.97)	1012	64.81 (20.65)	353	63.17 (20.35)	320	64.84 (20.28)	305	65.53 (20.66)
Family	1216	78.60 (16.46)	1150	79.28 (16.90)	951	78.81 (18.85)	1177	79.30 (16.35)	1101	77.51 (17.65)	1008	79.27 (16.70)	351	81.02 (14.79)	321	80.98 (16.13)	306	80.57 (16.69)
Friends	1210	70.60 (15.90)	1156	70.95 (16.66)	948	71.62 (17.04)	1168	71.10 (16.24)	1088	70.12 (16.40)	1010	71.76 (17.63)	348	70.04 (16.54)	319	71.10 (16.22)	304	72.13 (16.57)
School	1209	64.11 (18.08)	1158	63.11 (17.87)	955	63.08 (18.55)	1169	63.96 (18.43)	1100	60.43 (18.01)	1010	62.03 (18.98)	349	64.76 (15.98)	320	65.10 (17.51)	304	63.29 (17.55)
Boys																		
Physical well-being	594	74.00 (20.01)	561	74.18 (19.62)	458	75.20 (19.93)	554	74.43 (19.86)	511	72.27 (20.40)	472	75.84 (19.42)	170	73.82 (21.58)	152	77.30 (18.29)	150	77.00 (17.84)
Emotional well-being	609	76.11 (15.54)	558	75.37 (16.40)	464	74.57 (17.01)	560	76.68 (15.55)	514	72.72 (17.33)	484	75.88 (16.55)	171	77.92 (13.78)	152	77.84 (15.59)	151	76.70 (16.17)
Self-esteem	608	65.11 (20.03)	562	67.60 (20.33)	462	67.28 (20.41)	559	63.89 (20.09)	514	65.28 (20.23)	485	67.08 (20.71)	171	66.89 (18.75)	153	67.85 (20.53)	150	67.79 (21.33)
Family	608	77.16 (16.47)	559	77.52 (17.43)	463	75.69 (18.81)	558	77.98 (16.68)	512	74.54 (18.70)	483	77.62 (17.09)	170	80.58 (14.74)	153	79.93 (16.46)	151	79.66 (16.22)
Friends	603	69.52 (16.25)	560	69.03 (17.19)	460	68.18 (17.83)	557	69.83 (16.43)	510	67.30 (16.89)	482	69.24 (17.71)	168	71.24 (16.27)	152	71.03 (15.09)	150	71.78 (16.40)
School	603	65.50 (18.01)	562	64.61 (16.89)	463	64.22 (18.72)	554	65.43 (17.47)	513	61.94 (17.54)	483	64.17 (17.48)	169	68.31 (31.92)	153	67.78 (17.15)	151	66.80 (16.09)
Girls																		
Physical well-being	609	65.23 (22.88)	594	67.83 (21.54)	491	68.80 (20.09)	616	66.79 (22.33)	583	69.03 (20.25)	522	68.30 (21.57)	183	66.31 (22.58)	167	69.11 (20.91)	154	66.82 (22.22)
Emotional well-being	611	75.59 (15.93)	593	76.41 (14.91)	493	77.24 (14.58)	621	76.18 (15.10)	588	75.57 (15.11)	528	75.21 (16.53)	181	72.95 (16.86)	168	74.54 (17.01)	154	74.58 (15.06)
Self-esteem	608	60.57 (19.81)	592	62.62 (19.73)	491	63.97 (18.09)	620	60.55 (19.66)	586	62.51 (19.63)	527	62.72 (20.40)	182	59.67 (21.22)	167	62.08 (19.69)	154	63.34 (19.88)
Family	608	80.04 (16.33)	590	81.00 (16.18)	488	81.76 (16.37)	619	80.49 (15.97)	587	80.12 (16.24)	525	80.79 (16.21)	181	81.43 (14.86)	168	81.93 (15.81)	154	81.41 (17.19)
Friends	607	71.68 (15.49)	595	72.78 (15.94)	488	74.87 (15.58)	611	72.26 (15.99)	576	72.63 (15.58)	528	74.05 (17.25)	180	68.92 (16.75)	167	71.17 (17.23)	153	72.34 (16.76)
School	606	62.73 (18.06)	595	61.72 (18.66)	492	62.01 (18.34)	615	62.64 (19.17)	585	59.12 (18.35)	527	60.06 (20.08)	180	61.42 (17.07)	167	62.64 (17.53)	152	59.76 (18.31)

### Baseline measurements

There was no difference between mindfulness intervention, active control (i.e., relaxation), and inactive control group at baseline in HRQoL. The mean HRQoL for all participants was 69.72 (SD = 13.11), ranging from 13.54 to 100. Boys had higher HRQoL compared to girls at baseline (*β* = 1.249, 95% CI 0.408–2.090, *p* = 0.004). Mean scores by grades were 71.66 (SD = 13.07) for 6th grade, 69.91 (SD = 13.21) for 7th grade, and 68.05 (SD = 12.88) for 8th grade.

### Overall mindfulness intervention effects on HRQoL for all students

Table 2 shows the intervention effects on HRQoL adjusted for grade and gender. Results show a positive intervention effect between mindfulness intervention and the active control group at 9 weeks (Group × T9 *β* = 1.587, 95% CI 0.672–2.502, *p* < 0.001) due to slight increase in HRQoL scores in the intervention group and decrease in the control group. These effects wane at 26 weeks (*β* = 0.953, 95% CI − 0.221–2.127, *p* = 0.112).

**Table 2:** Results of multilevel models: intervention effects on KINDL total among all students, by gender, and by grade

All				Boys		Girls		Grade 6		Grade 7		Grade 8	
	Estimate	95% CI	*P* value	Estimate	95% CI	Estimate	95% CI	Estimate	95% CI	Estimate	95% CI	Estimate	95% CI
Change by 9 weeks													
Group (Int vs. 0) × T9	−0.958	(−2.303; 0.387)	0.163	−0.597	(−2.708; 1.514)	−1.348	(−3.008; 0.312)	−1.598	(−4.189; 0.993)	0.572	(−1.890; 3.034)	0.145	(−2.091; 2.381)
Group (Int vs. Control) × T9	**1.587**	**(0.672; 2.502)**	** < 0.001**	2.156^**^	**(0.719; 3.593)**	1.159^*^	**(0.032; 2.286)**	0.544	(−1.051; 2.139)	4.087***	**(1.692; 6.482)**	1.856**	**(0.660; 3.052)**
Change by 26 weeks													
Group (Int vs. 0) × T26	−0.159	(−1.841; 1.523)	0.853	−0.982	(−3.604; 1.640)	0.52	(−1.434; 2.474)	1.414	(−1.508; 4.336)	1.393	(−1.465; 4.251)	0.59	(−2.254; 3.434)
Group (Int vs. Control) × T26	0.953	(−0.221; 2.127)	0.112	−0.143	(−1.987; 1.701)	2.028^**^	**(0.685; 3.371)**	−0.307	(−2.087; 1.473)	3.498*	**(0.754; 6.242)**	1.686*	**(0.093; 3.279)**

*CI* confidence interval

Statistically significant (*p* < 0.05) estimates from Wald tests are bolded. Statistical significance of estimates: *p < 0.001; **p < 0.01; ***p < 0.05

### Mindfulness intervention effects by gender

Positive mindfulness intervention effects were found for HRQoL at 9 weeks for girls (*β* = 1.159, 95% CI 0.032–2.286, *p* = 0.044) and for boys (*β* = 2.156, 95% CI 0.719–3.593, *p* = 0.003) compared with the active control group. For boys the intervention effect was significant due to decreased HRQoL scores in the active control group. Gender modified the intervention effects on HRQoL at 26 weeks (group × gender × T26, *p* = 0.030). Positive intervention effects were found for HRQoL at 26 weeks for girls (*β* = 2.028, 95% CI 0.685–3.371, *p* = 0.003) compared with the active control group. There were no intervention effects for boys at 26 weeks (*β* = − 0.143, 95% CI − 1.987–1.701, *p* = 0.879). There were no gender differences in intervention attendance or in the amount of independent practice.

### Mindfulness intervention effects by grade

The effect of intervention was significantly different between 7 and 6th grade students at 9 weeks (group × grade × T9, *p* = 0.022), there were no other effects between grades. There were no significant intervention effects for 6th grade students. Positive intervention effects were found for 7th grade students at 9 weeks (*β* = 4.087, 95% CI 1.692–6.482, *p* < 0.001) and at 26 weeks (*β* = 3.498, 95% CI 0.754–6.242, *p* = 0.013), as well as 8th grade students at 9 weeks (*β* = 1.856, 95% CI 0.660–3.052, *p* = 0.002) and at 26 weeks (*β* = 1.686, 95% CI 0.093–3.279, *p* = 0.038) compared to the active control group.

### Intervention effects by independent practice

Table 3 shows the intervention effects on HRQoL adjusted for gender and grade by independent practice intensity. The intervention was found most effective for students who had practiced mindfulness nearly every day compared to the active control and inactive control group. Students with high frequency mindfulness practice had positive intervention effects in HRQoL at 9 weeks (*β* = 4.462, 95% CI 1.248–7.676, *p* = 0.007) and at 26 weeks (*β* = 5.441, 95% CI 2.058–8.824, *p* = 0.002) compared with the active control group and at 26 weeks (*β* = 4.333, 95% CI 0.742–7.924, *p* = 0.018) compared with the inactive control group. Descriptive statistics for different levels of practice are shown in Table 4.

**Table 3:** Results of multilevel models: intervention effects on KINDL total by practice intensity

	Estimate	95% CI
Change by 9 weeks		
Group (Int0 vs. 0) × T9	−1.092	(−2.527; 0.343)
Group (Int1 vs. 0) × T9	−1.775	(−4.135; 0.585)
Group (Int2 vs. 0) × T9	−1.233	(−4.385; 1.919)
Group (Int3 vs. 0) × T9	1.916	(−1.445; 5.277)
Group (Int0 vs. Control) × T9	1.453^**^	**(0.410; 2.496)**
Group (Int1 vs. Control) × T9	0.770	(−1.374; 2.914)
Group (Int2 vs. Control) × T9	1.313	(−1.682; 4.308)
Group (Int3 vs. Control) × T9	4.462^**^	**(1.248; 7.676)**
Change by 26 weeks		
Group (Int0 vs. 0) × T26	−0.309	(−2.055; 1.437)
Group (Int1 vs. 0) × T26	−1.642	(−4.300; 1.016)
Group (Int2 vs. 0) × T26	1.045	(−2.383; 4.473
Group (Int3 vs. 0) × T26	4.333^*^	**(0.742; 7.924)**
Group (Int0 vs. Control) × T26	0.800	(−0.462; 2.062)
Group (Int1 vs. Control) × T26	−0.533	(−2.901; 1.835)
Group (Int2 vs. Control) × T26	2.154	(− 1.055; 5.363)
Group (Int3 vs. Control) × T2	5.441^**^	**(2.058; 8.824)**

Statistically significant (*p* < 0.05) estimates from Wald tests are bolded

*CI* confidence interval

Practice intensity after the intervention was classified as follows:

*Int0* a couple of times during 6 months, *Int1 *once/twice a month, *Int2 * at least once a week, *Int3* nearly every day, *Control* Active control group, *0* = Non-treatment group

Statistical significance of estimates: *p < 0.001; **p < 0.01; ***p < 0.05

**Table 4:** Intervention dosage by group and subscale

Intervention dosage	Group	Subscale variable	Baseline	
Almost everyday	Intervention		N	Mean (SD)
		Physical well-being	40	68.65 (20.27)
		Emotional well-being	42	72.47 (18.83)
		Self-esteem	42	66.82 (21.76)
		Family	42	77.53 (17.08)
		Friends	42	66.57 (18.17)
		School	42	63.39 (l19.98)
At least once a week	Intervention			
		Physical well-being	47	69.95 (22.82)
		Emotional well-being	47	73.67 (15.36)
		Self-esteem	46	60.46 (20.75)
		Family	46	82.61 (14.25)
		Friends	46	66.03 (13.92)
		School	45	62.08 (17.18)
Once or twice a month	Intervention			
		Physical well-being	94	72.23 (20.76)
		Emotional well-being	97	76.76 (14.17)
		Self-esteem	95	65.53 (20.01)
		Family	96	78.75 (16.25)
		Friends	95	69.87 (14.69)
		School	95	64.41 (17.87)
Only few times	Intervention			
		Physical well-being	675	70.05 (21.43)
		Emotional well-being	682	76.51 (14.79)
		Self-esteem	681	62.90 (19.25)
		Family	682	79.05 (16.40)
		Friends	677	71.42 (15.38)
		School	678	64.96 (17.74)
No dosage	Active Control			
		Physical well-being	1170	70.41 (21.53)
		Emotional well-being	1181	76.42 (15.31)
		Self-esteem	1179	62.14 (19.92)
		Family	1177	79.30 (16.35)
		Friends	1168	71.10 (16.24)
		School	1169	63.96 (18.43)
No dosage	Non-treatment			
		Physical well-being	353	69.93 (22.39)
		Emotional well-being	352	75.37 (15.62)
		Self-esteem	353	63.17 (20.35)
		Family	351	81.02 (14.79)
		Friends	348	70.04 (16.54)
		School	349	64.76 (15.98)

### Effect sizes

The largest intervention effects on HRQoL were observed in the highest intensity practice group at 9 weeks (*d* = 0.35) and 26 weeks (*d* = 0.43) compared to the active control and at 26 weeks (*d* = 0.34) compared to the inactive control group (Table 5). The intervention increased HRQoL in the 7th grade students compared to the active control group at 9 weeks (*d* = 0.32) and at 26 weeks (*d* = 0.27).

**Table 5:** Effect sizes (95% confidence intervals) for intervention group compared to control and non-treatment groups at 9 weeks (T9) and 26 weeks (T26)

	Intervention vs. control		Intervention vs. non-treatment	
Outcome	T9	T26	T9	T26
HRQoL				
All	0.12 (0.04; 0.20)	0.07 (−0.01; 0.15)	−0.07 (−0.19; 0.04)	−0.01 (−0.13; 0.11)
Boys	0.17 (0.06; 0.29)	−0.01 (−0.13; 0.10)	−0.05 (−0.22; 0.12)	−0.08 (−0.25; 0.09)
Girls	0.09 (−0.02; 0.20)	0.16 (0.04; 0.27)	−0.10 (−0.27; 0.06)	0.04 (−0.13; 0.21)
Grade 6	0.04 (−0.09; 0.17)	−0.02 (−0.15; 0.11)	−0.13 (−0.33; 0.08)	0.11 (−0.09; 0.32)
Grade 7	0.32 (0.11; 0.53)	0.27 (0.06; 0.48)	0.04 (−0.18; 0.27)	0.11 (−0.11; 0.33)
Grade 8	0.15 (0.03; 0.26)	0.13 (0.02; 0.25)	0.01 (−0.21; 0.23)	0.05 (−0.17; 0.27)
The highest intensity	0.35 (0.04; 0.66)	0.43 (0.12; 0.74)	0.15 (−0.17; 0.47)	0.34 (0.02; 0.66)

Effect sizes (Cohen’s d) were calculated as the multilevel model adjusted intervention

Effect (group × T9 or group × T26) divided by the unadjusted pooled standard deviation at the baseline

Cohen *d* = 0.2 is considered as a ‘small’ effect size, *d* = 0.5 as a ‘medium’ effect size

## Discussion

This study aimed to examine whether a mindfulness-based intervention program shows unique effect on children’s self-reported health-related quality of life among 12–15-year-old students. We found that a mindfulness intervention vs. relaxation-based active control group has an immediate intervention effect on HRQoL for all participants that does not last to 26-week follow-up. However, when we examined the gender and grade effects, we found a significant improvement among girls and 7^th^ and 8th grade students at 9 weeks and at 26 weeks. We also discovered the frequency of independent practice was associated with higher HRQoL. It seems that the mindfulness intervention has the potential to improve general HRQoL in school children, but it does not impact all students equally.

### Mindfulness intervention has selective beneficial effects on HRQoL

During the past few years there have been a number of RCTs on the effectiveness of mindfulness interventions in schools [8, 17, 49]. The evidence base has strengthened from small trials, where numbers were too few to consider age and gender effects, and controls were mostly passive non-treatment groups, if at all existent. Research so far suggests that mindfulness training has small to moderate effect on psychological, subjective well-being among children and adolescents. However, there are some studies where this effect has waned [23, 26, 33] when similar control has been provided. In the present study, we found a significant effect for all participants in HRQoL after 9 weeks of intervention, due to decreased HRQoL in the active control group. There was no effect compared to the smaller inactive control group. The lack of effect compared to the inactive control may be due to insufficient size of the inactive group in cluster randomized research, as school-specific factors have greater potential to influence the results. As the effect was not consistently found at 26 weeks, we suggest that the mindfulness intervention may contribute to a short-term protective impact on HRQoL for all, but optimally would be supported by regular practice and enhanced by receptive gender or grade for long-term benefits.

### Mindfulness has significant gender-mediated effects on HRQoL

Mindfulness-based intervention increased girls’ HRQoL significantly compared to the control and non-treatment groups and the effect persisted from immediate post-intervention at 9 weeks to follow-up at 26 weeks. These results align with existing evidence of more pronounced effects among women [12, 13, 27] and girls [26].

There are affect-related risk factors that seem to be gender biased, i.e., females may be more prone to affective processing of negative emotions [35] and tend to engage in maladaptive coping strategies, e.g., rumination and self-critique [1, 9]. As mindfulness interventions address and improve positive affectivity, adaptive coping and self-compassion, as well as decrease rumination, they may be by nature more targeted toward girls [3, 41]. There were no gender differences in intervention attendance or in independent practice; thus, girls did not improve more by practicing or getting a higher dose of treatment.

There was a significant difference between the boys in intervention and active control group at 9 weeks. This was due to decreased HRQoL scores in the active control group and the effect did not persist in follow-up at 26 weeks. There was no significant difference between the boys in intervention and inactive control group at 9 weeks. Therefore, we suggest that as compared to the girls there is a more nuanced effect in boys, of which we can only speculate at this stage. It may be that the stress related to approaching end of term exams (at 9 weeks of follow-up end of term was 4–6 weeks away) might momentarily weaken the HRQoL in the active control group, whereas mindfulness-based training, which is designed to alleviate the stress and offer some strategies to cope with exam anxiety, helps preserve HRQoL. This might show as a short-term buffer effect in the intervention group.

### Mindfulness has significant grade-mediated effects on HRQoL

Our findings of significant grade-mediated effects may offer guidance in age-optimal delivery of mindfulness curriculum. Volanen et al. [44] found that 7th graders benefitted more from mindfulness intervention in terms of resilience and psychosocial well-being, compared with 6th and 8th graders. Our results are in line with this finding and suggest that grade-related differences may be connected to transition from lower to upper school and not only to grade as such. The beginning of upper elementary school brings on changes in physical environment, supporting adults, peers, and educational demands [6, 48]. Grade differences at baseline also show that older children have significantly lower HRQoL. Start of upper school at the age of 13 may be a particularly opportune time for mindfulness training to alleviate the age-related decline in HRQoL.

### Regular mindfulness practice boosts HRQoL

Our results indicate that the dosage of independent practice in the intervention group was related to the changes in HRQoL. Previous studies Stop and Breathe program have found varying rates of continuous (once a week or more) practice, from 20% [29] to 12% [23]. According to our findings 10% of students, who reported their level of mindfulness practice, were practicing once a week or more after 6 months. About 4% of students were practicing nearly every day after 6 months, and these students also reported the most significant gains in HRQoL. There were no differences in baseline HRQoL between different practice groups (Table 2).

## Practical implication and directions for future research

The results of this study shed more light to school-based mindfulness interventions and their benefits. We discovered that it is more beneficial for girls and older students to take part in a mindfulness program in terms of HRQoL. We conclude that mindfulness interventions offer positive outcomes for girls in risk of anxiety, depression, and poor HRQoL [14]. We also propose that the current mindfulness-based interventions are not as effective with boys as compared to girls, and further research is warranted on developing more gender-tailored approaches. Adherence to independent practice during and after the intervention is generally low [23], so it could be recommended that school-based regular practice would follow the intervention period to boost the training. For example, there is some evidence that intensive practice may reduce the significance of gender as a moderator, showing equal benefits for boys and girls [44]. Further research on student experience with mixed methods may also shed more light on motivating factors and possible challenges [11].

## Methodological considerations

Some limitations should be noted. The number of participants was reduced by drop-outs (attrition 5.9%, with no significant differences between intervention and control groups), which was expected considering drop-out rates in similar trials. Boys were more likely to drop out than girls (63.9% of drop-outs) and students whose mother language was other than Finnish/Swedish were also more likely to drop out (14.6% of drop-outs). Other differences were small and not significant. The inactive control group was smaller than the intervention and active control groups (*n* = 353) and the active control group (*n* = 1181) was slightly under the power calculations for primary outcomes (*n* = 1200 in each group), which reduces statistical power. We also note that we did not control the experience in mindfulness at baseline, allowing the students varying backgrounds. However, the participating schools had not received previous mindfulness training.

The strengths in this study are related to the strong research design. That is, the use of active and inactive control groups as well as post-intervention follow-ups. Furthermore, the analyses were statistically accounted for classroom and school effect, and we utilized an evidence-based mindfulness intervention (Stop and Breathe) as well as established HRQoL measure. Randomization was conducted at school level, which reduces the risk of contamination between groups. In addition, the participants were blinded regarding their selection to intervention or active control program.

## Conclusions

Our study indicated that mindfulness-based intervention improves HRQoL for girls and 7th and 8th graders, but the effects do not extend to both genders and all examined age groups. Girls are known to have poorer HRQoL in general and older students are facing a challenging change both in terms of adolescent development and social environment. These results highlight the possibilities of mindfulness-based training for students, who may have more need for adaptive emotional skills to cope in their teenage years. Noting that the effects of mindfulness training continued to grow for girls and regular practitioners at 26 weeks of follow-up, we could strengthen the promotive health care approach in schools by developing evidence-based and specific long-term strategies. As regular independent practice seems to benefit both genders, we should explore ways to promote opportunities for school-based practice and aim to follow up the effects beyond immediate assessments.

## Supplementary Information

Below is the link to the electronic supplementary material.

Supplementary file1 (PPTX 44 kb)
