# Supplementary material for: Effects of school-based mindfulness intervention on health-related quality of life: moderating effect of gender, grade, and independent practice in cluster randomized controlled trial
Source: Qual Life Res. 2021 Jun 24;30(12):3407–19. doi: 10.1007/s11136-021-02868-4 (PMC8602227; doi:10.1007/s11136-021-02868-4)
Supplement: Supplementary file 1 — Supplementary file1 (PPTX 44 kb) [file 11136_2021_2868_MOESM1_ESM.pptx]

## Slide 1
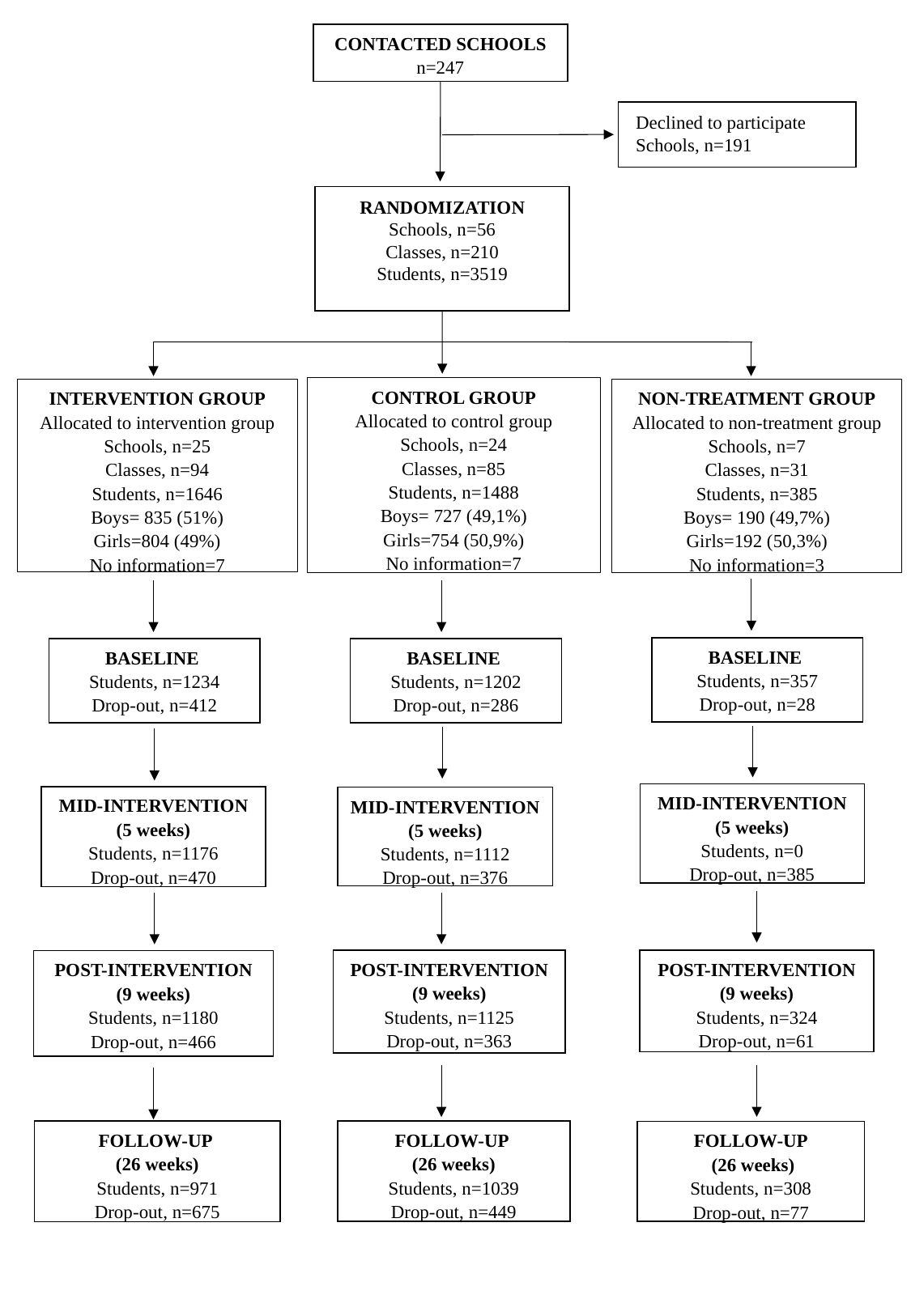

CONTACTED SCHOOLS
n=247
Declined to participate
Schools, n=191
RANDOMIZATION
Schools, n=56
Classes, n=210
Students, n=3519
CONTROL GROUP
Allocated to control group
Schools, n=24
Classes, n=85
Students, n=1488
Boys= 727 (49,1%)
Girls=754 (50,9%)
No information=7
NON-TREATMENT GROUP
Allocated to non-treatment group
Schools, n=7
Classes, n=31
Students, n=385
Boys= 190 (49,7%)
Girls=192 (50,3%)
No information=3
INTERVENTION GROUP
Allocated to intervention group
Schools, n=25
Classes, n=94
Students, n=1646
Boys= 835 (51%)
Girls=804 (49%)
No information=7
BASELINE
Students, n=357
Drop-out, n=28
BASELINE
Students, n=1202
Drop-out, n=286
BASELINE
Students, n=1234
Drop-out, n=412
MID-INTERVENTION (5 weeks)
Students, n=0
Drop-out, n=385
MID-INTERVENTION (5 weeks)
Students, n=1176
Drop-out, n=470
MID-INTERVENTION (5 weeks)
Students, n=1112
Drop-out, n=376
POST-INTERVENTION (9 weeks)
Students, n=1125
Drop-out, n=363
POST-INTERVENTION (9 weeks)
Students, n=324
Drop-out, n=61
POST-INTERVENTION (9 weeks)
Students, n=1180
Drop-out, n=466
FOLLOW-UP
(26 weeks)
Students, n=971
Drop-out, n=675
FOLLOW-UP
(26 weeks)
Students, n=1039
Drop-out, n=449
FOLLOW-UP
 (26 weeks)
Students, n=308
Drop-out, n=77
